# Improving Completion of Extended Discharge Documents (EDD) in a Forensic Psychiatry Service: A Baseline Audit and Reaudit Following System Change

**DOI:** 10.1192/bjo.2026.11815

**Published:** 2026-06-30

**Authors:** Kenechukwu Kennedy Orazulike, Gemma Fleming

**Affiliations:** NHS Grampian, Aberdeen, United Kingdom

## Abstract

**Aims:**

Extended Discharge Documents (EDD) are central to safe transitions of care in forensic psychiatry, providing GPs and community teams with information on treatment, risk management, legal status, and followup plans. National standards recommend issuing the EDD within 7–14 days of discharge. A baseline audit in March–August 2025 at the forensic unit in Royal Cornhill Hospital showed low EDD completion and variation between wards. Several service changes were introduced, and a reaudit was conducted. The aim was to assess whether these changes improved EDD completion and timeliness for forensic discharges.

**Methods:**

This was a retrospective clinical audit of all forensic inpatient discharges across two periods: the baseline cycle (March–August 2025) and the reaudit cycle (September 2025–January 2026). Patients transferred to other hospitals were excluded, as an EDD was not required for those cases. Data were collected from electronic records, including EDD eligibility, completion status, dispatch dates, and ward of discharge. The main outcome measures were the proportion of eligible discharges with a completed EDD, and the proportion completed within 14 days.

The intervention implemented between cycles included assigning each EDD to a named doctor at the point of discharge planning and reinforcing standards during induction for rotating trainees. A shared forensic handover template (digital and paper versions) was introduced to help track outstanding EDDs.

**Results:**

There were 42 discharge episodes in the baseline period, of which 41 required an EDD. Only 8 were completed (19.5%), and 1 of these (12.5%) was delayed beyond 14 days.

In the re-audit period, there were 29 discharge episodes, of which 27 required an EDD. Of these, 22 were completed (81.5%). Completion was consistently above 75% across all months, reaching 100% in January. The improvement was observed across most wards.

Timeliness remained a key concern. Sixteen of the 22 completed EDDs (72.7%) were issued more than 14 days after discharge, with higher delay rates noted in specific.

**Conclusion:**

Introducing system-level changes like assigning named doctors at discharge, strengthening induction, and introducing a shared handover template were associated with a marked improvement in overall EDD completion, rising from 19.5% to 81.5%. However, delays in issuing the EDD remain frequent and represent the key area for further improvement. Strengthening early drafting, adding timeliness checkpoints, and embedding automated reminders may help close this gap in the next audit cycle.

